# Association of estrogen receptor alpha gene polymorphism with age at onset, general psychopathology symptoms, and therapeutic effect of schizophrenia

**DOI:** 10.1186/1744-9081-9-12

**Published:** 2013-03-15

**Authors:** Shuai Wang, Wenqiang Li, Jingyuan Zhao, Hongxing Zhang, Yongfeng Yang, Xiujuan Wang, Ge Yang, Luxian Lv

**Affiliations:** 1 Department of Psychiatry, Henan Mental Hospital, The Second Affiliated Hospital of Xinxiang, Medical University, No.388, Jianshe Middle Road, Xinxiang, 453002, China; 2 Henan Key Lab of Biological Psychiatry, Xinxiang Medical University, No.388, Jianshe Middle Road, Xinxiang, 453002, China

**Keywords:** Estrogen receptor, Polymorphism, Association, Schizophrenia, Age at onset, Symptom, Therapeutic effect

## Abstract

**Background:**

Estrogen is believed to play an important role in the central nervous system (CNS) and exert a protective role against schizophrenia. Estrogen receptor alpha (ESRα) mediates the biological action of estrogen. Rs2234693 and rs9340799, single nucleotide polymorphisms of *ESRα*, may be related to many psychiatric disorders, while their association with schizophrenia has not been clarified.

**Methods:**

Genotypes rs2234693 and rs9340799 were detected in 303 schizophrenic patients and 292 healthy controls in a Chinese population. The positive and negative syndrome scale (PANSS) was used to estimate symptoms and therapeutic effects. The association of these polymorphisms with schizophrenia and clinical characteristics was analyzed by the chi-square test, analysis of variance, and others.

**Results:**

The distribution of genotypes and allele frequencies of rs2234693 and rs9340799 exhibited no significant differences between patients and controls, while haplotypes consisting of these polymorphisms had significant differences. For 2234693, T-allele carriers had an earlier age at onset. CC-homozygote carriers had a higher general psychopathology score and its percentage reduction in male and paranoid patients, respectively. CC-homozygote carriers had a higher tension (G4) and poor impulse control (G14) score, mainly in paranoid patients. Furthermore, patients with the CC homozygote had higher reductions of G4 and G14 scores when treated by aripirazole and risperidone, respectively.

**Conclusions:**

Haplotypes consisting of these two polymorphisms in *ESRα* may be strongly associated with schizophrenia. The rs2234693 was related to age at onset, general psychopathology, G4 and G14 symptoms, even the therapeutic effect in different groups.

## Background

Schizophrenia is a severe and polygenic inherited disease with an incidence of appropriately 1% worldwide [1]. The mechanism of its pathogenesis has been explored in various studies, but it is still undefined. Some psychiatric clinical data show the obvious differences between the two genders in age at onset, symptomatology, and prognosis for schizophrenia [2]. The peak age of onset for females is 4 to 6 years later than that of males [3]. Furthermore, affective symptoms are more prevalent in female patients [4], while functional impairment is more likely present in males [5]. In addition, female patients have a better overall prognosis than males [6].

The gender differences might be explained by the modern estrogen hypothesis, which presupposes that estrogen exerts a protective activity against schizophrenia [7]. The ovarian steroid hormone estrogen plays an important role in biological processes, and its influence on the CNS has been fully described [8,9]. The estrogenic level fluctuation was in different genders and ages, which has long been demonstrated [10,11]. On the other hand, patients with an older age at onset may live with a decreasing estrogenic level [10]. The cognitive abilities of patients with higher estrogenic levels are better than those with lower levels [12]. Furthermore, a previous study has found that the positive effect of estrogen treatment with standardized antipsychotic medication may be effective in replacement therapy for female schizophrenia [13].

The biological actions of estrogen are manifest through estrogen receptors, which belong to a large family of nuclear receptors [14]. Two estrogen receptors have been identified, designated as estrogen receptor alpha (ESRα) and estrogen receptor beta (ESRβ) [14]. *ESRα* localizes in chromosome 6 and encodes a 6.8-kilobase mRNA containing eight exons, finally becoming transformed into diverse proteins with different binding abilities [15], while *ESRβ* localizes in chromosome 14 and encodes a protein which is 97% and 59% homologous in the amino acid sequence with ESRα in DNA- and hormone-binding domains, respectively [16]. The distribution and biological activation of the two estrogen receptors in the nervous system are generally consistent [17], but ESRα appears to be responsible for most of the known estrogenic activations [18].

Recent studies have determined that the single nucleotide polymorphisms (SNPs) of *ESRα* might be related to many disorders, including osteoporosis [19], stroke [20], and Alzheimer’s disease [21]. Interestingly, other studies have indicated that the SNPs in *ESRα* are also associated with many psychiatric disorders. A case–control study showed a significant association of *ESRα* SNP rs2234693 with major depression disorder [22]. Furthermore, the *ESRα* haplotypes created by rs2234693 and rs9340799 polymorphisms increased the likelihood of having an anxiety disorder but not a depressive disorder [23]. Schizophrenia is a highly heterogeneous disease effected by multiple genes that generally belong to different hypothesis for the etiology of this disorder [24], such as the dopamine D2 receptor gene, a key member of dopamine hypothesis for schizophrenia, whose polymorphisms are associated with schizophrenia [25]. Based on the estrogen hypothesis, Weickert et al. [26] reported that rs2234693 was related to *ESRα* mRNA levels, associating with schizophrenia in their African American case–control sample, but other studies [27,28] had different point of view.

Overall, the conclusions of association between *ESRα* and schizophrenia are inconsistent. The purpose of this study was to determine whether there exists an association of *ESRα* polymorphisms with schizophrenia in our samples. Furthermore, we also examined the association of the polymorphisms with clinical characteristics including age at onset, symptoms, and therapeutic effects.

## Materials and methods

### Subjects

Three hundred three inpatients with schizophrenia (male/female = 152/151) were recruited from the Second Affiliated Hospital of Xinxiang Medical University. The patients were diagnosed in accordance with *Diagnostic and Statistical Manual of Mental Disorders,* Fourth Edition (DSM-IV) criteria by at least two experienced psychiatrists on the basis of structured clinical interviews and medical records. Patients were treated by monotherapy with aripiprazole (Abilify) (n = 146) or risperidone (Risperdal) (n = 157) after a washout period of 6 weeks. The average daily dose of antipsychotics for each patient was 4.9 ± 1.6 mg/day in risperidone equivalents.

Two hundred ninety-two healthy controls (male/ female = 146/146) without a psychiatric history (including personal and family) and complex disorders including diabetes, hypertension, immunological mediated disease, and tumors were selected from local Henan Province. All participators in the study were Chinese Hans. The mean age of the case group was 29.99 ± 8.86, and that of the control group was 30.92 ± 9.12. No significant differences in sex or age between the two groups existed.

All subjects consented to participate in the study after reviewing the informed consent. The study was approved by the Institutional Review Board of the Second Affiliated Hospital of Xinxiang Medical University.

### Clinical measures

The age at which the individual first experienced a diagnosis or symptoms of schizophrenia was defined as the age at onset of schizophrenia. To assess the psychopathologic severity of the patients, PANSS (including 3 subscales, positive symptom, negative symptom and general psychopathology symptom) was used by four experienced psychiatrists. The severity of each symptom was scored with one of seven grades (1 = absent, 2 = minimal, 3 = mild, 4 = moderate, 5 = moderate severe, 6 = severe, 7 = extreme). The PANSS scores at week 0 were regarded as baseline. To evaluate the therapeutic effect over 6 weeks, the percentage reduction in PANSS score and reduction in PANSS subscore were used. All the psychiatrists had been trained in the use of PANSS before the study.

### Genotyping

Global genomic DNA was extracted from the peripheral leucocytes using standard protocols. The *ESRα* fragment, including rs2234693 and rs9340799 polymorphisms, was amplified by the polymerase chain reaction (PCR) with the following primers: Sense: 5^′^-CCTTTCTGTGTTCCTCTTCT-3^′^; antisense: 5^′^-TACCTCTTGCCGTCTGTT-3^′^. PCR amplification was performed in a 25-μL reaction volume containing 10 × PCR buffer (supplied by Tian Gen) 2.5 μL, dNTP mix (2.5 mM, supplied by Tian Gen) 0.5 μL, sense primer (10 μM, supplied by Genewiz) 1 μL, antisense primer (10 μM, supplied by Genewiz) 1 μL, genomic DNA 1 μL, Taq DNA polymerase (5 U/μL, supplied by Tian Gen) 0.4 μL, and sterile deionized water 18.6 μL. After initial denaturation at 94°C for 5 min, the ingredients were mixed at 35 cycles at 94°C for 30 s, 55°C for 30 s, 72°C for 54 s, and a final elongation was done at 72°C for 10 min. The PCR product was cleaved using *Pvu II* and *Xba I* restriction enzymes (supplied by Thermo) at 37°C for 2 h, then analyzed by electrophoresis on 1.5% agarose gels. Genotypes for rs2234693 and rs9340799 polymorphisms were identified by two investigators independently.

### Statistical analysis

Hardy Weinberg equilibrium was performed using Haploview 4.1 to examine the genotype distributions of these two polymorphisms of *ESRα*. The strength of linkage disequilibrium between the two SNPs was determined using D’ and r^2^ algorithm testing by SHEsis online software (http://analysis2.bio-x.cn/myAnalysis.php) [29]. The Kaplan-Meier method and the log-rank test for analysis of survival were used to examine the association of age at onset with the two polymorphisms [30]. The difference in genotypic and allelic frequency distribution between case and control groups was estimated by SPSS software 18.0 using the Pearson chi-square (*χ*^*2*^) test. Haplotype analysis for the *ESRα* was performed using SNPStats software on line (http://bioinfo.iconcologia.net/snpstats) [31]. The relationship between the polymorphisms and the clinical variables was also analyzed by SPSS 18.0 using one-way ANOVA or rank sum test. In addition, to control for the possible confounding effect of gender and therapeutic drugs, stratification of the samples was repeated by those factors, respectively [32]. The Bonferroni correction was applied to avoid a type I error in the multiple tests. A power analysis was performed using the Genetic Power Calculator [33]. All statistical analyses were two-tailed, and the level of statistical significance was adjusted to *p* < 0.05.

## Results

### Genotype identification

The PCR products were 899 base pairs. The three genotypes resulting from digestion with *Pvu II* were CC (899 bp), CT (899, 630, 269 bp), and TT (630, 269 bp). Similarly, three genotypes were yielded from digestion with *Xba I*: GG (899 bp), AG (899, 675, 224 bp), and AA (675, 224 bp). The codominant model was a combination of separate genotypes of CC, CT, and TT for rs2234693, and AG, GG, and GG for rs9340799. The dominant model consisted of a C-allele carrier and TT homozygote for rs2234693, and a G-allele carrier and AA homozygote for rs9340799. Furthermore, the recessive model was formed by a T-allele carrier and CC homozygote for rs2234693, and an A-allele carrier and GG homozygote for rs9340799.

### Association of SNPs with schizophrenia

The distributions of genotypes and allele frequencies of the *ESRα* polymorphisms for all, male, and female groups were in Hardy-Weinberg equilibrium. The genotype and allele distribution of rs2234693 had no significant differences when comparing cases and controls, even in the subgroups stratified by gender (Table 1). No significant differences were demonstrated for the genotype and allele distribution of rs9340799 in comparing cases and controls. But when stratified according to gender, a nominal difference was found for genotype distribution and a significant difference for allele distribution in male cases with schizophrenia and male controls (corrected *p* = 0.05 and *p* = 0.02, respectively), but no significant differences when comparing for females (Table 1). This study had the power of 0.739 overall.

**Table 1:** Association analysis of SNPs with schizophrenia

**dbSNP ID**	**Group**	**Cases**						**Controls**						***p*-value**	
		**N**	**HWE ( *p *)**	**Genotype**			**MAF**	**N**	**HWE ( *p *)**	**Genotype**			**MAF**	**Genotype**	**Allele**
rs2234693				TT	TC	CC				TT	TC	CC			
	All	303	0.55	109	150	44	0.15	292	0.71	111	141	40	0.14	0.868	0.612
	Male	152	0.86	63	71	18	0.12	146	0.46	60	71	15	0.10	0.894	0.876
	Female	151	0.51	46	79	26	0.17	146	1.00	51	70	25	0.17	0.692	0.574
rs9340799				AA	AG	GG				AA	AG	GG			
	All	303	1.00	194	97	12	0.04	292	0.85	189	93	10	0.03	0.938	0.789
	Male	152	0.50	91	51	10	0.07	146	0.53	104	40	2	0.01	0.025	**0.010**
	Female	151	0.37	103	46	2	0.01	146	1.00	85	53	8	0.05	0.057	0.031

Boldface letters represent that the Bonferroni corrected *p*-values are still less than 0.05.

### Association of haplotypes with schizophrenia

**Figure 1:**
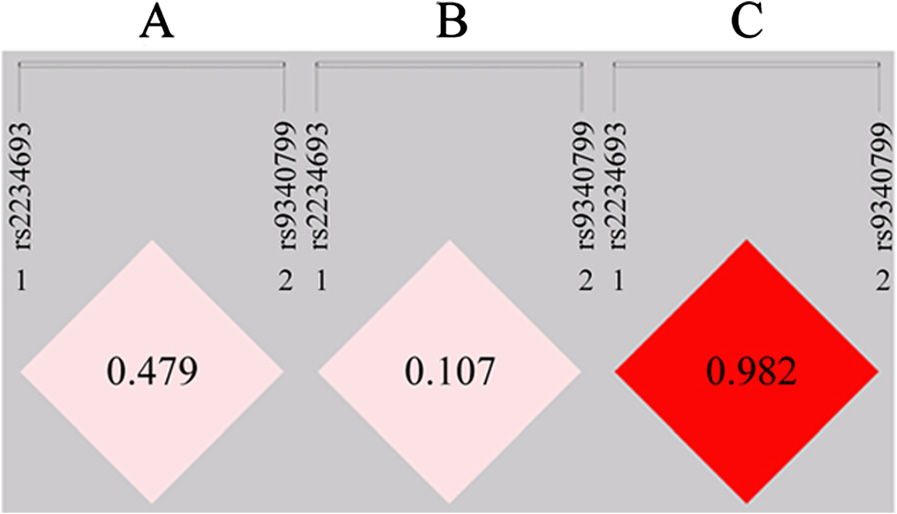
**Linkage disequilibrium among rs2234693 and rs9340799. A**, linkage disequilibrium in all samples with 0.479 for D’; **B**, linkage disequilibrium in patients with 0.107 for D’; **C**, linkage disequilibrium in controls with 0.982 for D’.

In all samples, a modest linkage disequilibrium was observed between rs2234693 and rs9340799 (D’ = 0.479, r^2^ = 0.089). A strong linkage disequilibrium was found in the analysis of normal controls (D’ = 0.982, r^2^ = 0.380), but not for patients with schizophrenia (D’ = 0.107, r^2^ = 0.002) (Figure 1). A haplotype was constructed from rs2234693 and rs9340799. Four haplotypes were recognized after combining with the two polymorphisms. T-A was the most prevalent haplotype, with frequencies of 0.5502, 0.5633, and 0.5403 in all, male, and female subjects, respectively, followed by the C-A, C-G, and T-G haplotypes (Table 2). In all and female subjects, the frequencies of C-A, C-G, and T-G showed a significant difference between cases and controls, whereas in the male subjects, only C-A was significantly different (corrected *p* = 0.0036). In addition, significant associations of global haplotype with schizophrenia showed up in both males and females (corrected *p <* 0.0001).

**Table 2:** The rs2234693-rs9340799 haplotype association analysis

**Group**	**rs2234693**	**rs9340799**	**Frequencies**			**OR (95% CI)**	***p*-value**	**Global haplotype association**
			**Cases**	**Controls**	**All**			
All	T	A	0.478	0.620	0.550	1		**< 0.0001**
	C	A	0.323	0.187	0.253	0.45 (0.33 - 0.61)	**< 0.0001**	
	C	G	0.070	0.191	0.133	1.94 (1.23 - 3.06)	**0.0046**	
	T	G	0.130	0.002	0.064	0.01 (0.00 - 0.10)	**< 0.0001**	
Male	T	A	0.473	0.654	0.563	1		**< 0.0001**
	C	A	0.294	0.195	0.244	0.47 (0.30 - 0.73)	**0.0009**	
	C	G	0.058	0.151	0.105	1.75 (0.83 - 3.69)	0.14	
	T	G	0.176	0.000	0.088	0.00 (−Inf - Inf)	1	
Female	T	A	0.490	0.585	0.540	1		**< 0.0001**
	C	A	0.344	0.179	0.259	0.44 (0.28 - 0.68)	**0.0002**	
	C	G	0.090	0.232	0.163	2.04 (1.18 - 3.53)	**0.012**	
	T	G	0.076	0.004	0.037	0.04 (0.01 - 0.35)	**0.0035**	

Boldface letters represent that the Bonferroni corrected *p*-values are still less than 0.05.

### Age at onset analysis

**Figure 2:**
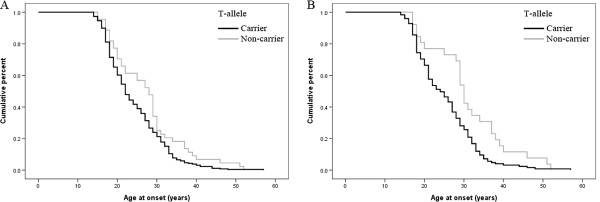
**The Kaplan-Meier plot showing the age at onset of schizophrenia with T allele. A**, the earlier age at onset in all cases carrying the T allele of rs2234693; **B**, the earlier age at onset in female cases carrying the T allele of rs2234693.

The rs2234693 polymorphism may be related to age at onset in the association analysis of *ESRα* and initial clinical features of schizophrenia (*p* = 0.0089) (Figure 2A). The ages at onset of T-allele carriers and those of noncarriers were 24.2 ± 7.4 and 27.5 ± 9.1, respectively. When stratified by gender, the association was more significant in the female cases (*p* = 0.001, 25.2 ± 6.2, and 31.0 ± 9.8 years for the T-allele carriers and noncarriers, respectively) (Figure 2B), but not in the male cases (*p* = 0.663). For rs9340799, no significant association was found in any groups.

### Association of SNPs with clinical symptoms

Of 201 patients with schizophrenia enrolled in this analysis, the distributions of rs2234693 (CC = 31, CT = 99, and TT = 71) and rs9340799 (AA = 133, AG = 60 and GG = 8) genotypes were in Hardy-Weinberg equilibrium (*p* = 0.77 and *p* = 0.65, respectively). Clinical symptoms, including the baseline of total, positive symptom, negative symptom, and general psychopathology symptom were analyzed in this study first. There was no significant difference in total, positive, or negative scores when comparing diverse genotypes of rs2234693 (data not shown). However, in comparing the general psychopathology scores, the difference was significant among TT (40.49 ± 5.81), TC (43.22 ± 5.37), and CC (45.58 ± 7.09) genotypes of rs2234693 only in male patients with schizophrenia (Table 3). Furthermore, the subscores of general psychopathology symptoms were examined in all stratified patients. The CC genotype carriers of rs2234693 had much higher scores (3.35 ± 1.56) than T-allele carriers (TC, 2.40 ± 1.45; TT, 2.39 ± 1.47) in poor impulse control (G14), and the difference still existed in the paranoid patients (Table 3). In addition, the tension (G4) of general psychopathology subscores was significant higher in the CC (3.21 ± 1.53) genotype carriers than in the T-allele carriers (TC, 2.55 ± 1.47; TT, 2.18 ± 1.30), only in the paranoid patients (Table 3). None of the clinical variables of the baseline symptoms was significantly different between the various genotypes of rs9340799 (Additional file 1: Table S1).

**Table 3:** Association analysis of rs2234693 with the base line of symptoms

**Group**	**Characteristic**	**Codominant**		**Dominant**		**Recessive**	
		***F*/*χ***^***2***^	***p*-value**	***F*/*χ***^***2***^	***p*-value**	***F*/*χ***^***2***^	***p*-value**
All	General psychopathology score	0.671	0.512	0.186	0.666	1.345	0.248
	Tension score	2.407	0.093	0.031	0.859	4.561	0.034
	Poor impulse control score	5.506	**0.005**	1.132	0.289	11.065	**0.001**
Male	General psychopathology score	4.345	**0.016**	1.476	0.228	4.303	0.041
	Tension score	0.915	0.405	0.011	0.915	1.733	0.191
	Poor impulse control score	3.367	0.039	0.073	0.788	5.505	0.021
Female	General psychopathology score	0.543	0.582	1.069	0.303	0.025	0.875
	Tension score	1.469	0.235	0.007	0.934	2.763	0.099
	Poor impulse control score	3.458	0.035	3.244	0.074	5.558	0.020
Paranoid	General psychopathology score	1.430	0.242	0.035	0.853	2.762	0.098
	Tension score	5.208	**0.006**	0.341	0.560	7.968	**0.005**
	Poor impulse control score	5.613	**0.004**	0.718	0.398	11.268	**0.001**

Boldface letters represent that the Bonferroni corrected *p*-values are still less than 0.05.

### Association of SNPs with therapeutic effect

The associations of rs2234693 with general psychopathology, tension (G4), and poor impulse control (G14) score of PANSS have been verified above. Thus, we further investigated whether rs2234693 is related to improvement in these symptoms after 6 weeks of antipsychotic treatment. Thereby, the percentage reduction of general psychopathology score was higher in the C-allele carriers than TT-homozygote carriers in female (CC+CT, 0.456 ± 0.270; TT, 0.327 ± 0.216) and paranoid (CC+CT, 0.464 ± 0.241; TT, 0.367 ± 0.213) patients with schizophrenia (Table 4). Furthermore, the reduction of G14 was higher in the CC-homozygote carriers than in the C-allele carriers overall (CC, 1.900 ± 1.795; CT+TT, 1.020 ± 1.287), male (CC, 2.170 ± 2.125; CT+TT, 1.040 ± 1.272), and paranoid (CC, 2.000 ± 1.888; CT+TT, 0.990 ± 1.289) patients with schizophrenia (Table 4). In addition, to control for possible confounding effects of the therapeutic drug, the patients were stratified by antipsychotic drug. Interestingly, the CC-homozygote carriers (1.800 ± 1.265) showed a greater G4 reduction than did the CT (0.900 ± 1.357) and TT (0.550 ± 0.905) genotype carriers after treatment by aripirazole, while CC-homozygote carriers (2.380 ± 1.628) had more G14 reduction than did CT (1.350 ± 1.336) and TT (0.860 ± 1.332) genotype carriers after treatment by risperidone (Table 4). None of the clinical variables of the reductions was significantly different between genotypes of rs9340799 (Additional file 2: Table S2).

**Table 4:** Association analysis of rs2234693 with therapeutic effects in 6-week therapy

**Group**	**Characteristic**	**Codominant**		**Dominant**		**Recessive**	
		**F/X**^**2**^	***p*-value**	**F/X**^**2**^	***p*-value**	**F/X**^**2**^	***p*-value**
All	Percentage reduction in general psychopathology score	2.964	0.054	5.259	0.023	0.003	0.955
	Reduction in tension score	1.879	0.155	0.693	0.406	3.718	0.055
	Reduction in poor impulse control score	1.928	0.165	4.153	0.043	10.721	**0.001**
Male	Percentage reduction in general psychopathology score	0.290	0.749	0.508	0.478	0.246	0.621
	Reduction in tension score	1.482	0.233	0.599	0.441	2.938	0.090
	Reduction in poor impulse control score	0.157	0.692	0.330	0.567	6.657	**0.012**
Female	Percentage reduction in general psychopathology score	3.784	0.026	6.168	**0.015**	0.123	0.727
	Reduction in tension score	0.562	0.572	0.148	0.701	1.129	0.290
	Reduction in poor impulse control score	3.946	0.022	5.604	0.020	4.514	0.036
Paranoid	Percentage reduction in general psychopathology score	3.264	0.041	6.567	**0.011**	0.636	0.426
	Reduction in tension score	2.339	0.100	0.223	0.637	4.670	0.032
	Reduction in poor impulse control score	1.163	0.281	3.442	0.065	10.756	**0.001**
Aripirazole	Percentage reduction in general psychopathology score	1.321	0.272	2.316	0.132	1.148	0.287
	Reduction in tension score	5.721	**0.005**	4.920	0.029	9.731	**0.002**
	Reduction in poor impulse control score	1.019	0.365	0.538	0.465	1.989	0.162
Risperidone	Percentage reduction in general psychopathology score	2.213	0.115	1.797	0.183	1.274	0.262
	Reduction in tension score	0.591	0.555	1.136	0.289	0.295	0.588
	Reduction in poor impulse control score	6.632	**0.002**	6.035	**0.016**	10.423	**0.002**

Boldface letters represent that the Bonferroni corrected *p*-values are still less than 0.05.

## Discussion

We conclude that the *ESRα* rs2234693 and rs9340799 polymorphisms do not substantially contribute to susceptibility of schizophrenia, although a modest association was detected in males only for rs9340799. The results are nearly consistent with most previous studies. This difference may be due to these reasons: on one hand, the subjects in the study of Weickert were African American [26] only, while others were East Asians [28,34] or southern Europeans [27]. Susceptibility to the disease and distribution of the genotype for rs2234693 were distinctly different in these subjects (data was from International HapMap Project). On the other hand, the sample size and test power between these studies were difference. Interestingly, the haplotypes in the gene may increase the risk of schizophrenia. C-A rarely emerges with the extremely high frequency of approximately 25%, and it may contribute to schizophrenia. It is noteworthy that C-G may play the role of protective haplotype, especially in females. It is also meaningful that T-G and C-G may act only on females, not males.

The age of onset has been considered the single most valuable characteristic of schizophrenia that may yield a clue to its origin [30]. A genomewide linkage study by Cardno et al. [35] has confirmed a genetic contribution to the age at onset of schizophrenia. This was the first time that significant associations of *ESRα* polymorphisms with age at onset of schizophrenia have been demonstrated. But in the study by Ouyang et al. [34], this association was not clearly verified. This inconsistency might be due to differences in population and in the definition of age at onset. In the present study, it is interesting that the T-allele of rs2234693 may relate to earlier pathogenesis of schizophrenia in all and female patients, not in males independently. Evidence that female schizophrenics are more easily influenced by inheritance than males may contribute to the gender difference [36].

Although many genes do not alter susceptibility to schizophrenia, they may affect clinical features [37,38]. A study by Alonso et al. [32] verified that rs34535804 and five SNP haplotypes in ESRα were associated only with the psychopathic symptoms contamination obsessions and cleaning compulsions. In our study, we hypothesize that all, male, and paranoid schizophrenic patients with the T-allele had generally poor impulse control, as well as both tension and poor impulse control symptoms, respectively, suggesting an association of these psychopathic symptoms with rs2234693 of *ESRα* in special populations. In the therapeutic effect analysis, female and paranoid patients with the CC homozygote had a superior therapeutic effect in general psychopathology symptom after 6 weeks. Notably, we found that aripriazole was more effective in treating tension symptoms in patients with the CC homozygote, while risperidone controlled poor impulse symptoms, again, in patients having the CC homozygote. These findings suggest that T-allele protects against the onset of these symptoms, especially in general psychopathology, tension, and poor-impulse control psychopathic symptoms. Furthermore, CC-homozygote carriers seem to be more sensitive to antipsychotic treatment. It is also worth noting that two polymorphisms are closely located on the ESRα gene, but this study has demonstrated that rs2234693 is not linked in disequilibrium with rs9340799 in patients with schizophrenia. This explains why only rs2234693 without rs9340799 is associated with clinical characteristics of schizophrenia.

ESRα is markedly different from other neurotransmitter receptors in that it may influence mood, cognition, and synaptogenesis and be involved in neuroprotective effects [9,39]. ESRα, as a ligand-dependent transcription factor, is widely distributed in the amygdala-hippocampal area, periamygdaloid cortex, and posterior cortical nucleus of the brain [40], especially in several limbic structures, suggesting its relationship with the above-mentioned processes [41]. Furthermore, in the CNS, ESRα binds its specific ligand estrogen to influence various neurotransmitter systems such as dopamine, serotonin (5-HT), and norepinephrine. These phenomena may be responsible for the relation between ESRα and many psychiatric disorders such as schizophrenia [40]. A previous review by Herrington et al. [42] has summarized the associations of *ESRα* polymorphisms with mood and cognition dysfunction, suggesting a strong relationship between them. We conclude that the association mechanism of *ESRα* variants with psychiatric characteristics may involve the estrogen-estrogen receptor effect, above. Whether our results can explain this mechanism needs further study.

In this study, we found a significant association of *ESRα* polymorphism with age at onset, general psychopathology symptoms, and, for the first time, the therapeutic effects in schizophrenia, but limitations also existed. Firstly, as only two polymorphisms were selected for analysis, they do not represent the whole gamut of the human *ESRα*, further studies should be done to analyze more variants. Secondly, not all the patients were included in the association analysis of clinical variables due to the finite clinical data. Thirdly, because the structured clinical interviews were difficult and time-consuming, our sample size was inadequate and could easily have led to false positive or negative results. Fourthly, although stratified analyses were performed, more subtle and stratified analyses should be used in further studies. Finally, whether *ESRα* variants can be considered as genetic markers to determine the risk or prognosis of schizophrenia needs further exploration.

## Conclusions

Our findings suggest that the haplotypes consisting of rs2234693 and rs9340799 in *ESRα* are mainly associated with schizophrenia. The rs2234693 polymorphism is related to the age at onset of schizophrenia. Notably, *ESRα* may affect the general psychopathology symptoms and its improvement in specific patients with schizophrenia. Additionally, we noticed that tension and poor impulse control symptoms were improved more significantly in CC-homozygote carriers after treatment by aripirazole and risperidone, respectively.

## Abbreviations

CNS: Central nervous system; ESRα: Estrogen receptor alphap; PANSS: Positive and negative syndrome scale; ESRβ: Estrogen receptor beta; SNP: Single nucleotide polymorphism; PCR: Polymerase chain reaction.

## Competing interests

The authors declare that they have no competing interests.

## Authors’ contributions

Author SW, WL, and LL designed the study. SW wrote the protocol and the first draft of the manuscript. Author SW and XW finished the biological experiments. Author WL managed the literature searches and analyses. Author XS and HZ undertook the statistical analysis. Author JZ, YY, and GY collected clinical data. All authors contributed to and have approved the final manuscript.

## Supplementary Material

Additional file 1: Table S1 Association analysis of rs9340799 with the base line of symptoms. Click here for file

Additional file 2: Table S2 Association analysis of rs9340799 with therapeutic effects in 6-week therapy. Click here for file
