# Supplementary material for: Association of estrogen receptor alpha gene polymorphism with age at onset, general psychopathology symptoms, and therapeutic effect of schizophrenia
Source: Behav Brain Funct. 2013 Mar 15;9:12. doi: 10.1186/1744-9081-9-12 (PMC3608973; doi:10.1186/1744-9081-9-12)
Supplement: Additional file 1: Table S1 — Association analysis of rs9340799 with the base line of symptoms. [file 1744-9081-9-12-S1.docx]

## Supplement Table 1 Association analysis of rs9340799 with the base line of symptoms

| Group | Characteristic | Codominant | |  | Dominant | |  | Recessive | |
| --- | --- | --- | --- | --- | --- | --- | --- | --- | --- |
|  |  | *F*/*χ^2^* | P-value |  | *F*/*χ^2^* | P-value |  | *F*/*χ^2^* | P-value |
| All | Total score | 1.685 | 0.188 |  | 0.001 | 0.992 |  | 3.097 | 0.080 |
|  | Positive score | 1.403 | 0.248 |  | 0.052 | 0.821 |  | 2.334 | 0.128 |
|  | Negative score | 0.246 | 0.782 |  | 0.160 | 0.690 |  | 0.447 | 0.505 |
|  | General psychopathology score | 0.814 | 0.444 |  | 0.083 | 0.773 |  | 1.631 | 0.203 |
| Male | Total score | 1.509 | 0.227 |  | 0.041 | 0.840 |  | 2.875 | 0.094 |
|  | Positive score | 0.834 | 0.438 |  | 0.051 | 0.822 |  | 1.245 | 0.268 |
|  | Negative score | 1.335 | 0.269 |  | 2.246 | 0.138 |  | 1.303 | 0.257 |
|  | General psychopathology score | 0.440 | 0.645 |  | 0.000 | 0.992 |  | 0.786 | 0.378 |
| Female | Total score | 0.216 | 0.806 |  | 0.074 | 0.786 |  | 0.285 | 0.594 |
|  | Positive score | 0.230 | 0.795 |  | 0.082 | 0.775 |  | 0.304 | 0.584 |
|  | Negative score | 0.645 | 0.527 |  | 1.090 | 0.299 |  | 0.430 | 0.513 |
|  | General psychopathology score | 0.366 | 0.694 |  | 0.044 | 0.835 |  | 0.737 | 0.393 |
| Paranoid | Total score | 1.815 | 0.166 |  | 0.124 | 0.726 |  | 2.928 | 0.089 |
|  | Positive score | 0.411 | 0.664 |  | 0.028 | 0.867 |  | 0.821 | 0.366 |
|  | Negative score | 0.651 | 0.523 |  | 0.001 | 0.982 |  | 1.201 | 0.275 |
|  | General psychopathology score | 0.944 | 0.391 |  | 0.020 | 0.887 |  | 1.644 | 0.202 |
