# Supplementary material for: Association of estrogen receptor alpha gene polymorphism with age at onset, general psychopathology symptoms, and therapeutic effect of schizophrenia
Source: Behav Brain Funct. 2013 Mar 15;9:12. doi: 10.1186/1744-9081-9-12 (PMC3608973; doi:10.1186/1744-9081-9-12)
Supplement: Additional file 2: Table S2 — Association analysis of rs9340799 with therapeutic effects in 6-week therapy. [file 1744-9081-9-12-S2.docx]

## Supplement Table 2. Association analysis of rs9340799 with therapeutic effects in 6-week therapy

| Group | Characteristic | Codominant | |  | Dominant | |  | Recessive | |
| --- | --- | --- | --- | --- | --- | --- | --- | --- | --- |
|  |  | *F*/*χ^2^* | P-value |  | *F*/*χ^2^* | P-value |  | *F*/*χ^2^* | P-value |
| All | Percentage reduction in total score | 0.929 | 0.397 |  | 1.682 | 0.196 |  | 0.002 | 0.967 |
|  | Percentage reduction in positive score | 0.975 | 0.379 |  | 1.912 | 0.168 |  | 0.033 | 0.855 |
|  | Percentage reduction in negative score | 0.146 | 0.864 |  | 0.012 | 0.914 |  | 0.229 | 0.633 |
|  | Percentage reduction in general psychopathology score | 1.509 | 0.224 |  | 2.748 | 0.099 |  | 0.001 | 0.971 |
| Male | Percentage reduction in total score | 1.360 | 0.507 |  | 0.953 | 0.332 |  | 0.338 | 0.563 |
|  | Percentage reduction in positive score | 1.032 | 0.361 |  | 1.980 | 0.163 |  | 0.035 | 0.853 |
|  | Percentage reduction in negative score | 0.011 | 0.989 |  | 0.007 | 0.932 |  | 0.007 | 0.931 |
|  | Percentage reduction in general psychopathology score | 0.793 | 0.456 |  | 1.462 | 0.230 |  | 0.592 | 0.444 |
| Female | Percentage reduction in total score | 1.298 | 0.277 |  | 0.639 | 0.426 |  | 1.459 | 0.230 |
|  | Percentage reduction in positive score | 0.224 | 0.799 |  | 0.248 | 0.620 |  | 0.118 | 0.732 |
|  | Percentage reduction in negative score | 0.698 | 0.500 |  | 0.052 | 0.820 |  | 1.197 | 0.276 |
|  | Percentage reduction in general psychopathology score | 1.837 | 0.164 |  | 1.238 | 0.268 |  | 1.675 | 0.198 |
| Paranoid | Percentage reduction in total score | 0.395 | 0.674 |  | 0.681 | 0.411 |  | 0.011 | 0.917 |
|  | Percentage reduction in positive score | 0.719 | 0.489 |  | 1.441 | 0.232 |  | 0.155 | 0.694 |
|  | Percentage reduction in negative score | 0.098 | 0.907 |  | 0.009 | 0.926 |  | 0.196 | 0.659 |
|  | Percentage reduction in general psychopathology score | 0.739 | 0.479 |  | 1.080 | 0.300 |  | 0.111 | 0.739 |
| Aripirazole | Percentage reduction in total score | 1.040 | 0.358 |  | 1.649 | 0.202 |  | 0.939 | 0.335 |
|  | Percentage reduction in positive score | 0.943 | 0.393 |  | 0.942 | 0.334 |  | 1.420 | 0.237 |
|  | Percentage reduction in negative score | 0.525 | 0.593 |  | 0.983 | 0.324 |  | 0.000 | 0.986 |
|  | Percentage reduction in general psychopathology score | 1.229 | 0.298 |  | 2.292 | 0.134 |  | 0.639 | 0.426 |
| Risperidone | Percentage reduction in total score | 0.586 | 0.558 |  | 0.041 | 0.839 |  | 0.904 | 0.344 |
|  | Percentage reduction in positive score | 0.974 | 0.381 |  | 0.529 | 0.469 |  | 0.819 | 0.367 |
|  | Percentage reduction in negative score | 0.483 | 0.618 |  | 0.917 | 0.341 |  | 0.275 | 0.601 |
|  | Percentage reduction in general psychopathology score | 0.552 | 0.578 |  | 0.258 | 0.613 |  | 0.516 | 0.474 |
